# Willingness to pay for social health insurance in Ethiopia: A systematic review and meta-analysis

**DOI:** 10.3389/fpubh.2023.1089019

**Published:** 2023-03-22

**Authors:** Ewunetie Mekashaw Bayked, Husien Nurahmed Toleha, Beletu Berihun Chekole, Birhanu Demeke Workneh, Mesfin Haile Kahissay

**Affiliations:** ^1^Department of Pharmacy, College of Medicine and Health Sciences (CMHS), Wollo University, Dessie, Ethiopia; ^2^Department of Pharmacy, Woldia Comprehensive Specialized Hospital, Woldia, Ethiopia; ^3^Department of Pharmaceutics and Social Pharmacy, School of Pharmacy, College of Health Sciences, Addis Ababa University, Addis Ababa, Ethiopia

**Keywords:** willingness to pay, social health insurance, associated factors, systematic review, meta-analysis, Ethiopia

## Abstract

**Background:**

Ethiopia plans to introduce social health insurance with the aim of giving recipients high-quality, long-term universal health care. It was anticipated to be fully operational in 2014. However, due to strong opposition from public employees, the implementation has been delayed multiple times. As a result, more and more studies have been conducted to collect evidence about the issue. However, there is no national pooled evidence regarding the willingness to pay for the scheme. Thus, this review aimed to evaluate the willingness to pay for social health insurance and associated factors in Ethiopia.

**Methods:**

On September 1, 2022, database searches were conducted on Scopus, Hinari, PubMed, Google Scholar, and Semantic Scholar. Based on this search, 19 studies were included in the review. The risk of bias for the included studies was assessed using Joana Briggs Institute checklists. The data were extracted using Microsoft Excel. RevMan-5 was used to conduct the meta-analysis. The effect estimates assessed were the odds ratios at a *p*-value <0.05 with a 95% CI using the random effect model.

**Results:**

The pooled willingness to pay for social health insurance was 42.25% and was found to be affected by sociodemographic, health and illness status, health service related factors, awareness or knowledge level, perception or attitude toward the scheme, and factors related to the scheme. The pooled result showed that the willingness of participants to pay for the scheme was 16% less likely (OR = 0.84; 95% CI: 0.52–1.36). When the outlier was unchecked, the willingness to pay became 42% less likely (OR = 0.58; 95% CI: 0.37–0.91). The lowest willingness to pay for the scheme was in the Oromia region, while the highest was in Harar. Professionally, teachers were 7.67 times more likely to pay for the scheme (OR = 3.22; 95% CI: 1.80–5.76) than health professionals (OR = 0.42; 95% CI: 0.19–0.93).

**Conclusion:**

The willingness to pay for social health insurance was low, <50%, particularly among health professionals, which urges the Ethiopian health insurance service to deeply look into the issue.

## Introduction

Wellbeing requires good health (1). The economic success of a nation is inextricably linked to the health of its citizens. An effective and fair healthcare system is critical for breaking the vicious cycle of poverty and illness (2). Healthcare systems are concerned not just with improving people's health but also with shielding them from the financial consequences of illness. The goal for governments in low-income nations is to minimize the regressive burden of out-of-pocket (OOP) health-care payments by extending prepayment programs, which share financial risk and lower the probability of catastrophic health-care costs (3).

Risks could be pooled in a variety of ways, including through national (single payer), social, private, and community-based schemes (4). Through cross-subsidization, social health insurance (SHI) improves equitable access to quality health services (4, 5). Because if there is a large pool of insured people, the wealthy and healthy could subsidize the costs incurred by the poor and sick. As such, if premium payments are based on a percentage of monthly salary or on contributions related to income, high-income individuals will pay more into the scheme (4). This could be possible for the planned Ethiopian SHI, for which the premium is set at 3% of the monthly salary (5).

SHI is a risk-pooling method of financing and managing health care. It considers both the public's health hazards and the contributions of individuals, households, enterprises, and the government. As a result, it shields people from financial and health burdens while also being a fairly equitable means of paying for health care. However, despite their best efforts, many least-developed and low-middle-income nations have not been able to expand SHI coverage to the extent that is desired (6).

This could be due to the fact that the contribution to the scheme is partly influenced by the willingness to pay (WTP) of the individuals. The WTP is a stated preference that refers to the valuation of benefits in monetary terms for health-related commodities or services (7). It is determined by contingent valuation (CV), a method that uses survey methodologies to assess the benefit or worth of a program to individuals (8). To determine the WTP using CV, two general elements, a hypothetical scenario and the bidding vehicle, should be included. A hypothetical scenario is a description of the program that gives the respondents a detailed explanation of the good or service they are being asked to pay for. The bids, on the other hand, can be obtained in a number of ways, including through open- or closed-ended questions, a bidding game, or a payment card (9).

In 2010, Ethiopia issued a proclamation for SHI, with the goal of providing beneficiaries with high-quality, long-term universal health coverage (UHC) by pooling risks and lowering financial barriers at the point of service delivery (5). In 2013, the country passed regulations to introduce SHI by the following year (10), i.e., it was expected to be completely operational by 2014. However, the implementation has been postponed several times, owing to significant opposition from public employees (11). As a result, more and more research has been conducted to get evidence for this issue. Despite this much effort, there is no comprehensive pooled data regarding the WTP for SHI. Thus, this review aimed to evaluate the extent of the WTP for SHI and associated factors in Ethiopia.

## Methods

### Protocol and registration

The protocol for this review was registered in PROSPERO (https://www.crd.york.ac.uk/prospero/display_record.php?ID=CRD42022355933), and amendments were being made during the review process. The “Preferred Reporting Items for Systematic Reviews and Meta-Analyses (PRISMA) 2020 statement: an updated guideline for reporting systematic reviews” was used as a framework for all sections of the review (12) (Supplementary material 1).

### Eligibility criteria

Analytical, prevalent, and retrospective cross-sectional studies, as well as mixed study designs, were included. All published studies in English, both in community and institutional settings, and on the WTP for SHI among the formal sector in Ethiopia were considered. The following study parameters were also used to decide which studies to include: outcome variables, population (study units), year of the study, context (regions), sample size, and response rate.

All other studies with incomplete data and a high risk of bias were excluded. If a study had both published and unpublished copies with identical reports, the unpublished copy was excluded. Furthermore, if a study was published in more than one journal, it was considered a duplicate, and the most recently published one was chosen to be included in the review.

### Information sources and search strategy

On September 1, 2022, database searches were conducted on Scopus, Hinari, PubMed, Google Scholar, and Semantic Scholar (Supplementary material 2). Resources from PubMed and Hinari were searched manually. However, Scopus, Google Scholar, and Semantic Scholar have been searched using the “Perish or Publish” database searching tool version 8 (13). Registries like the Ethiopian Health Insurance Service (EHIS) and the general web were also searched for additional information. The databases were searched using text words and indexed terms, which included “willingness to pay,” “social health insurance,” “factors,” and “Ethiopia. “Additional filters were also employed: year of study, publication year, content type, discipline, and language. To find more relevant studies, the reference lists of studies that met the inclusion criteria were searched.

### Selection process

After duplicates and irrelevant studies were excluded using “Zotero” reference manager version 6, two reviewers, EMB and HNT, screened the included studies independently. The selection of studies was carefully screened by these two researchers. First, the articles were refined by their title and abstract; second, by full-text revision by these authors, independently and finally together, until reaching consensus. When disagreements arose, a third reviewer was contacted to resolve the difference. Then, all studies that fulfilled the eligibility criteria and had a score of “low” or “medium” risk of bias were included.

### Data collection process and data items

A Microsoft Excel spreadsheet was used for data extraction. Two reviewers, EMB and HNT, independently extracted the data, compared their conclusions, and came to an agreement. If not, a third reviewer was invited to help these two reviewers reach consensus. Moreover, the authors of the studies were contacted to collect the missing information.

The outcome variable, the population (study units), the year of study, the context, the sample size, the response rate, and funding sources were extracted by the excel spreadsheet. The main outcome of this review was WTP for SHI. The additional outcomes were the factors affecting the WTP for SHI.

### Study risk of bias assessment

The risk of bias for the included studies was assessed independently by two reviewers, EMB and HNT, using the Joanna Briggs Institute (JBI) critical appraisal checklists. The bias was assessed on: the criteria for inclusion in the sample; the description of study participants and setting; the validity and reliability of measurement; confounding and strategies to deal with it; and the appropriateness of the outcome measure. Accordingly, studies with a score of 7 or higher were labeled as low risk, 5–6 medium risk, and 4 or lower high risk. Then, those studies with low and medium risk were included in the review. Any inconsistencies were resolved by discussion and involving a third reviewer, as necessary.

### Effect measures

Prevalence, proportion, inverse variance, and odds ratios were calculated for each study. For the summary effect, the *X*^2^, *z*-value, *p*-value with a 95% CI, and odds ratio were computed.

### Synthesis methods

We used thematic analysis for the qualitative synthesis. Coming to the quantitative part, first, data (such as events, non-events, participants, and sample sizes) were extracted from each study using a Microsoft Excel spreadsheet. Then, preliminary effect measures, like the prevalent rate and proportion, as well as odds ratios of WTP for SHI, were computed in the spreadsheet. Finally, a generic inverse variance analysis was employed to estimate the overall effect sizes using Revman-5. The summary odds ratio with 95% CI was computed based on the random effect model. Sub-group analyses were conducted to compare the effect estimates across studies based on region (context) and profession. The level of overall statistical significance was determined using a *p*-value < 0.05 with a 95% CI.

### Reporting bias assessment

Reporting bias was assessed by considering whether the studies were published or not. It was also examined by the year of studies and the publication years of them. For those studies with incomplete or missing data, the study authors were contacted. The studies with incomplete data were excluded.

### Certainty assessment

The *I*^2^ statistic was used to evaluate between-study heterogeneity. The influence of each study on the overall meta-analysis was measured using inverse variance (percentage of weight). The funnel plot was used to examine the possibility of bias between studies (publication bias). Sensitivity analysis was performed by unchecking outlier studies.

## Results

### Study selection

**Figure 1:**
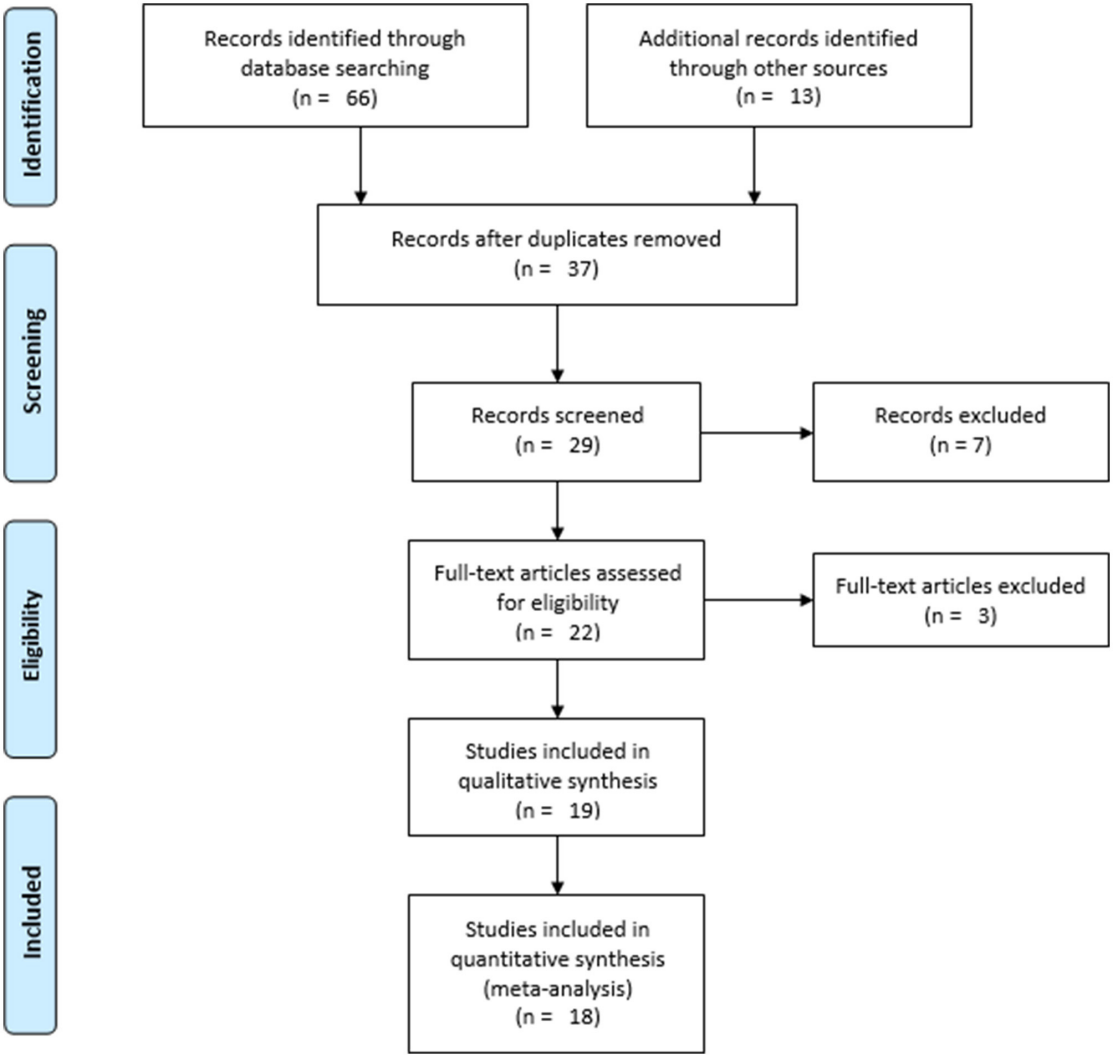
PRISMA flow diagram showing the selection processes of the included studies.

In total, 79 resources were identified (Figure 1). Sixty-six of them were identified from databases: Scopus (*n* = 7), Hinari (*n* = 21), PubMed (*n* = 11), Google Scholar (*n* = 17), and Semantic Scholar (*n* = 10). The remaining 13 sources were obtained from registries (*n* = 2) and websites (*n* = 11). Thirty-seven studies were identified after duplicates (*n* = 42) were removed. Following the exclusion of eight studies based on relevance, 29 studies were found to be eligible for title and abstract evaluation. Through title and abstract review, 22 studies were chosen to be eligible for full text evaluation. After one and two studies were removed due to incomplete data (14) and a high risk of bias (15, 16), 19 studies were included for the qualitative synthesis. Eighteen studies were then included for the quantitative synthesis after one was excluded due to its report of the WTP for SHI being below the set premium (3%) (17).

### Study characteristics

Most of the included studies were conducted in the Amhara region (*n* = 6), followed by Addis Ababa (*n* = 6), Tigray (*n* = 3), SNNPR (*n* = 2), Oromia (*n* = 1), and Harar (*n* = 1). In total, the sample population of the included studies was 9,325, of which 9,084 (97.42%) were found to be actual participants. The summary result of the individual study characteristics is shown in Table 1.

**Table 1:** Characteristics of the individual included studies, Ethiopia (*n* = 19), 2022.

**Study**	**Design**	**Area**	**Year**	**Outcome**	**SS**	**RR**	**Event**	**Prop**.	**Quality**
Degie et al. (18)	Cross-sectional	Amhara	2016	WTP	375	361	136	0.376	8/8
Gidey et al. (19)	Mixed	Tigray	2017	WTP	384	381	325	0.853	5/8
Mekonne et al. (20)	Cross-sectional	Addis Ababa	2019	WTP	460	445	128	0.287	7/8
Setegn et al. (21)	Cross-sectional	Amhara	2018	WTP	574	546	339	0.62	8/8
Agago et al. (22)	Cross-sectional	SNNPR	2012	WTJ & WTP	335	328	244	0.744	7/8
Yeshiwas et al. (23)	Cross-sectional	Amhara	2013	WTJ & WTP	557	488	325	0.666	8/8
Lasebew et al. (24)	Cross-sectional	Addis Ababa	2016	WTP	420	409	70	0.17	7/8
Mekonnen et al. (25)	Cross-sectional	Amhara	2018	WTJ & WTP	619	605	113	0.187	6/8
Gessesse et al. (26)	Cross-sectional	Tigray	2018	WTP	843	843	299	0.355	5/8
Tewele et al. (27)	Cross-sectional	Tigray	2017	WTJ & WTP	408	396	185	0.467	7/8
Regassa et al. (28)	Cross-sectional	Oromia	2018	WTJ &WTP	280	275	76	0.276	6/8
Mulatu et al. (29)	Cross-sectional	SNNPR	2019	WTJ & WTP	713	692	42	0.06	5/8
Kokebie et al. (30)	Cross-sectional	Addis Ababa	2020	WTP	506	503	178	0.354	7/8
Amilaku et al. (31)	Cross-sectional	Amhara	2021	WTP	845	796	236	0.296	8/8
Hailu et al. (32)	Cross-sectional	Harar	2021	WTP	323	272	243	0.893	5/8
Tadele et al. (33)	Cross-sectional	Addis Ababa	-	WTP	383	368	89	0.242	5/8
Hizkiyas (34)	Cross-sectional	Addis Ababa	2020	WTP	423	398	307	0.771	7/8
Salilew (17)	Cross-sectional	Amhara	2021	WTJ & WTP	597	698	674	0.965	8/8
Banti et al. (35)	Cross-sectional	Addis Ababa	2022	WTP	280	280	208	0.743	8/8
**Total**					9,325	9,084	4,217	0.4642	6.7/8

DCE, Discrete Choice Experiment; RR, Response rate; SNNPR, Southern Nations, nationalities and Peoples Region; SS, Sample Size.

### Risk of bias in studies

**Figure 2:**
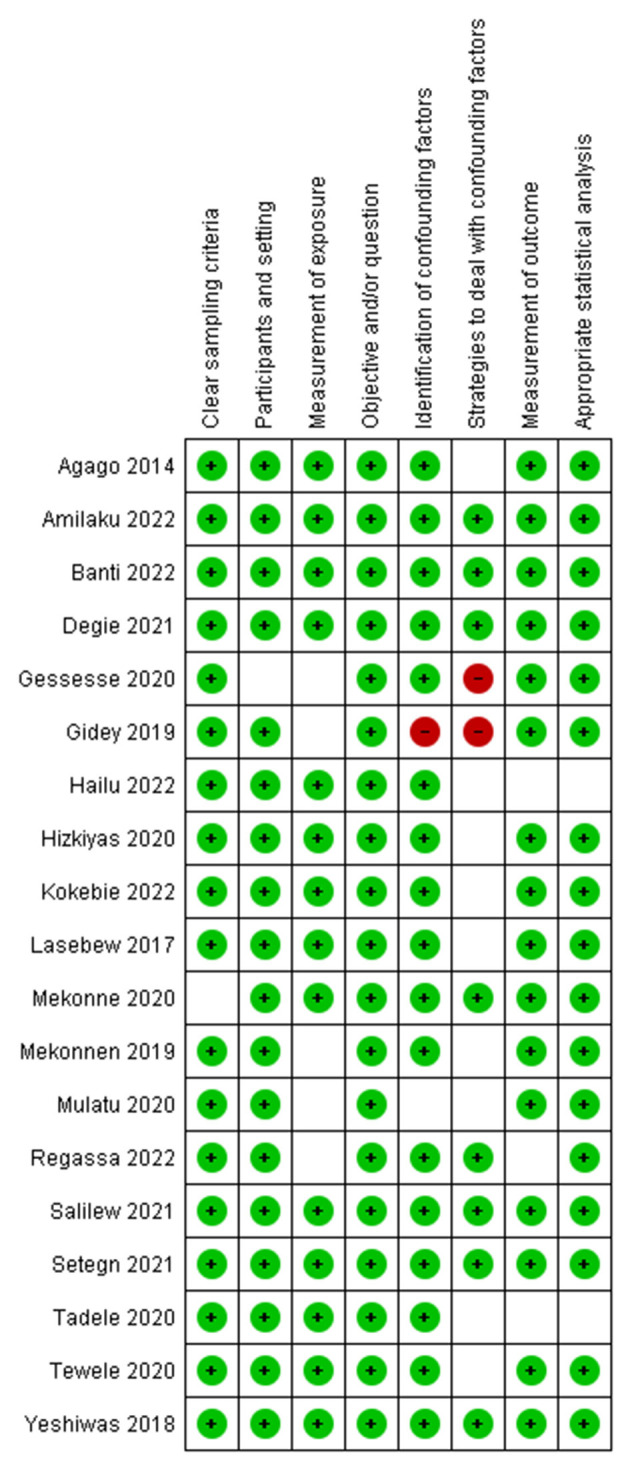
Summary of the risk of bias assessment of the included studies.

After the risk of bias for the included studies was assessed using JBI's critical appraisal tools, those studies with a low or medium risk were included in the study (Figure 2).

### Results of qualitative synthesis

The WTP for SHI was found to be influenced by sociodemographic factors like education level (20–22, 25, 31, 32), income (17, 19–21, 27, 28, 31, 34), age (26, 28, 31), marital status (28, 33), occupation or job description (28), family size (29, 30, 34), and job experience (16); health and illness status such as self-rated healthiness (16), the presence of acute (20, 21, 31), and chronic illnesses (34); health service related factors like previous medical bills (16, 18, 21, 22, 24, 27, 28, 30, 35), referral system (26), regular medical checkup (26), and health service quality (16, 19, 24–27); awareness or knowledge (16–18, 20–23, 27, 29, 30, 32, 34, 35); perception (24, 31); attitude (17, 21, 27, 33, 34); and factors related to the scheme such as trust (17, 23), premium amount (19), and the scope of the benefit packages (19, 25).

### Results of quantitative synthesis

A total of 9,084 participants were found from all 19 included studies, of which 38.46%, 26.45%, 17.83%, 11.23%, 3.03%, and 3.00% were in Amhara, Addis Ababa, Tigray, SNNPR, Oromia, and Harar, respectively (Table 1). However, for the quantitative synthesis, 18 studies with 8,386 participants were included, of whom 3,543 were willing to pay for the scheme, which provided a pooled WTP of 42.25% for SHI (Table 2).

**Table 2:** Pooled result of the meta-analysis by region.

**Subgroup**	**Studies**	**Participants**	**Events**	**Percent**	**Statistical method**	**Effect estimate**
Amhara	5	2,796	1,149	41.10	Odds ratio (IV, Random, 95% CI)	0.72 [0.33, 1.58]
Tigray	3	1,620	809	49.94	Odds ratio (IV, Random, 95% CI)	1.40 [0.43, 4.58]
Addis Ababa	6	2,403	980	40.78	Odds ratio (IV, Random, 95% CI)	0.72 [0.30, 1.73]
SNNPR	2	1,020	286	28.04	Odds ratio (IV, Random, 95% CI)	0.43 [0.01, 18.06]
Oromia	1	275	76	27.64	Odds ratio (IV, Random, 95% CI)	0.38 [0.29, 0.50]
Harar	1	272	243	89.34	Odds ratio (IV, Random, 95% CI)	8.38 [5.71, 12.31]
**Pooled result**	**18**	**8,386**	**3,543**	**42.25**	**Odds ratio (IV, Random, 95% CI)**	**0.84 [0.52, 1.36]**

**Figure 3:**
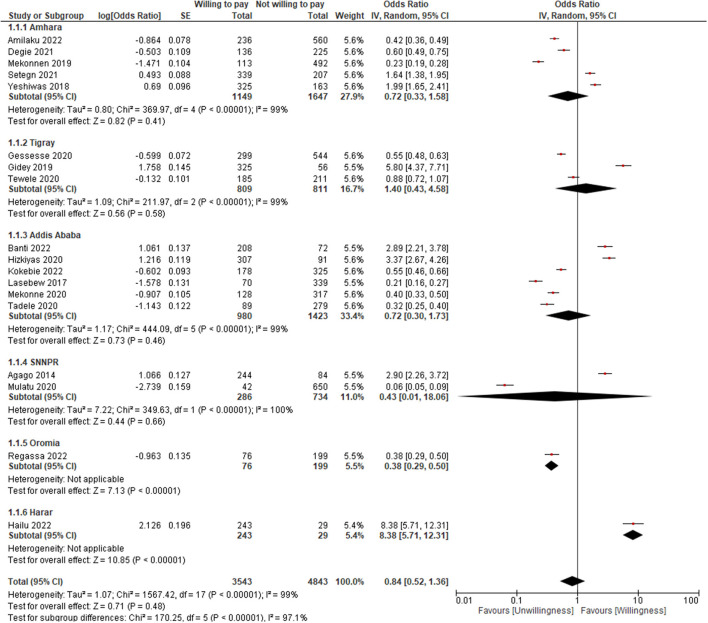
The forest plot before adjusted for outlier.

**Figure 4:**
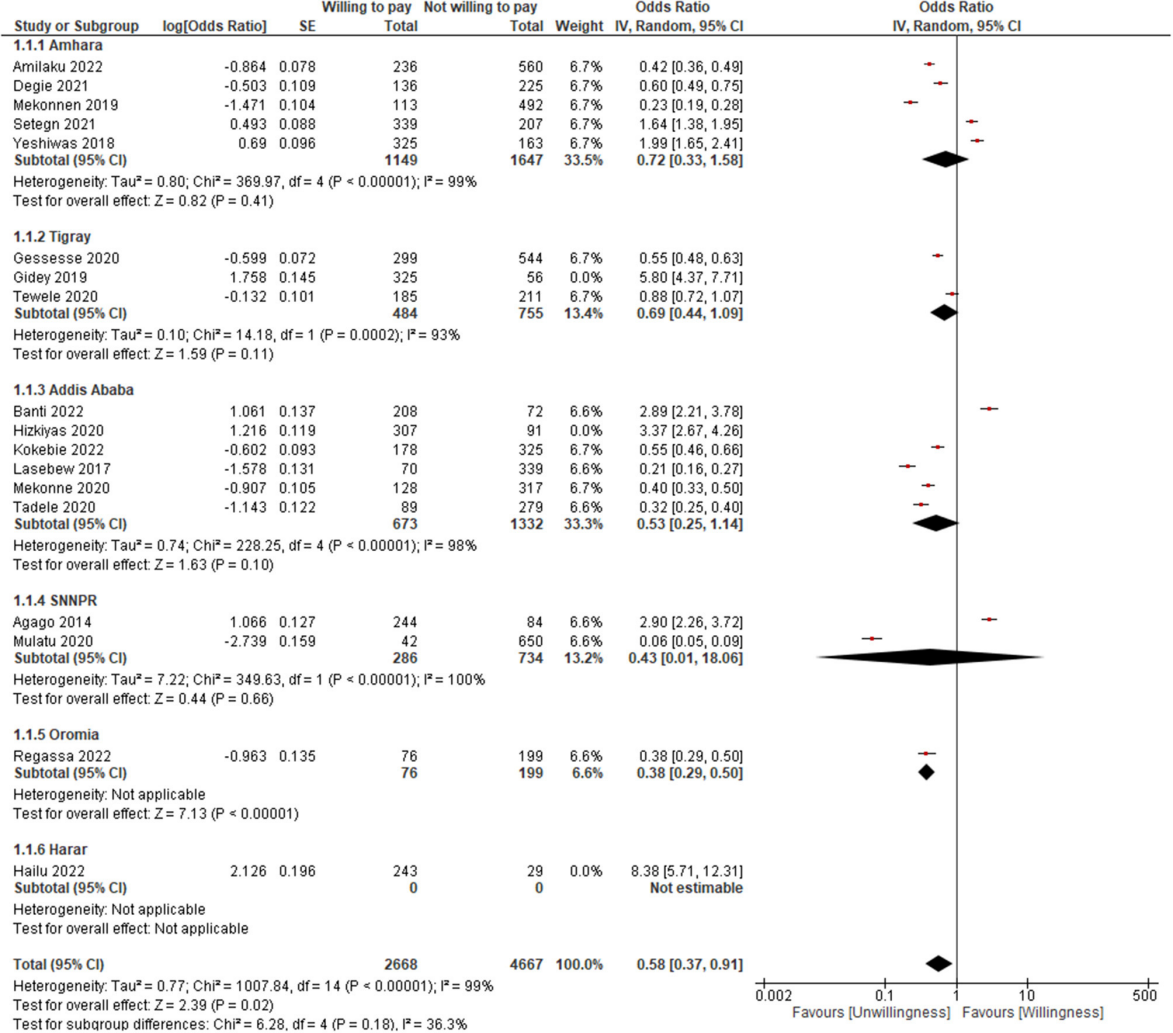
The forest plot after adjusted for outlier.

As indicated in Table 2 and Figure 3, the pooled result showed that the WTP for SHI was found to be 16% less likely and was not found to be significant (OR = 0.84; 95% CI: 0.52–1.36). The WTP was not also found to be significant for the following sub-groups: Amhara (OR = 0.72; 95% CI: 0.33–1.58), Tigray (OR = 1.40; 95% CI: 0.43–4.58), Addis Ababa (OR = 0.72; 95% CI: 0.30–1.73), SNNPR (OR = 0.43; 95% CI: 0.01–18.06), but for Oromia (OR = 0.38; 95% CI: 0.29–0.50) and Harar (OR = 8.38; 95% CI: 5.71–12.31). However, when the outlier was unchecked (32), it became significant (OR = 0.58; 95% CI: 0.37–0.91) (Figure 4). The lowest WTP for the scheme was in Oromia, while the highest was in Harar.

**Figure 5:**
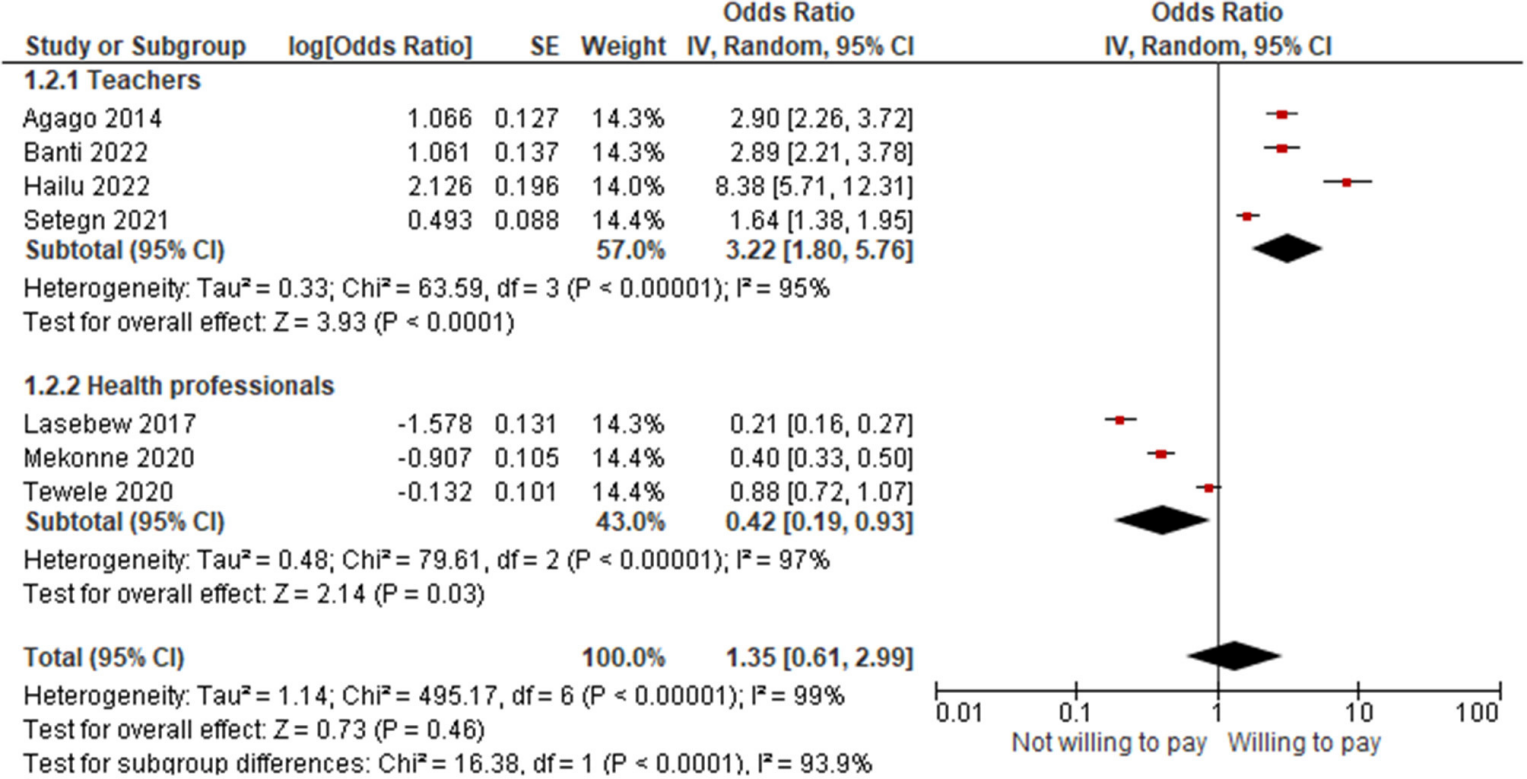
The forest plot for the sub-group analysis by profession.

We found that seven studies were conducted on homogeneous populations: teachers and health professionals. Thus, as shown in Figure 5, regarding the sub-group analysis by profession, teachers were 7.67 times more likely to pay for SHI (OR = 3.22; 95% CI: 1.80–5.76) than health professionals (OR = 0.42; 95% CI: 0.19–0.93).

### Reporting bias

The reports of the included studies for the factors affecting the WTP for SHI were not consistent. So, it was difficult to determine the direction of association for both the qualitative and quantitative synthesis.

### Certainty of evidence

**Figure 6:**
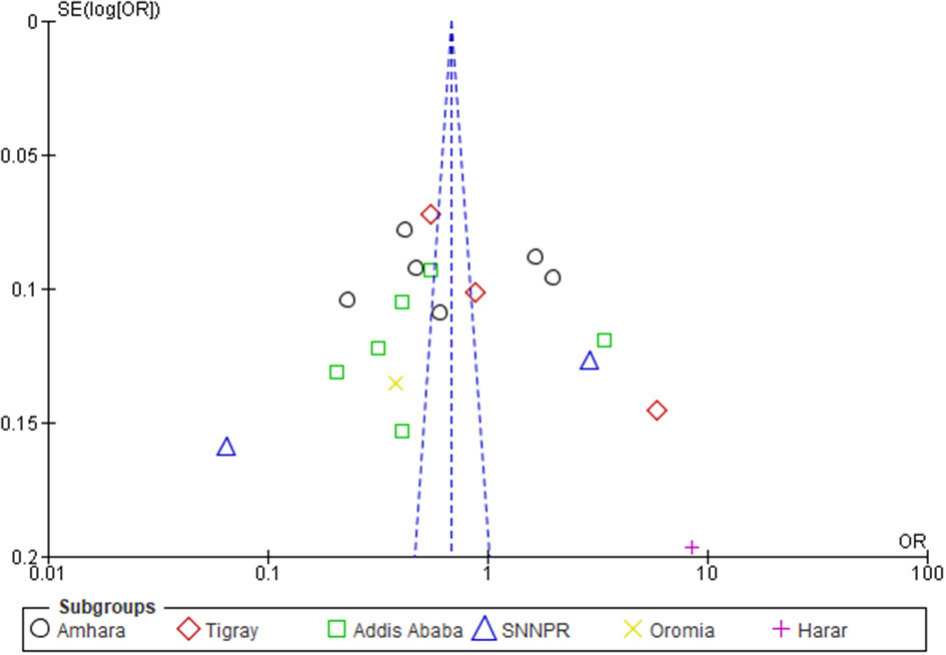
The summary analysis of publication bias.

The *I*^2^-values of the sub-group analyses were 93% to 100%, which are indicators of substantial heterogeneity (36). Thus, since the *I*^2^-value was >50%, a random-effect model was used to pool the WTP for SHI with a 95% CI (37). Through **s**ensitivity analysis, a study (32) was found to be an outlier, though the heterogeneity was not changed much (Figure 6).

## Discussion

The review revealed that the pooled WTP for SHI was 42.25% and was found to be affected by sociodemographic factors like education level, income, age, marital status, occupation or job description, family size, and job experience; health and illness status, such as self-rated healthiness and the presence of acute and chronic illnesses; health service-related factors like previous medical bills, referral systems, regular medical checkups, and health service quality; awareness, knowledge, perception, and attitude; and factors related to the scheme, such as trust, premium amount, and scope of the benefit packages.

The WTP for the scheme in Ethiopia was found to be less than the findings in Uganda (38), Indonesia (39), Nigeria (40), Bangladesh (41), Saudi Arabia (42), South Sudan (43), and Nepal (44), which reported that the WTP for the scheme was 91%, 87.36%, 82%, 80.10%, 76%, 52%, and 51%, respectively. The low level of the WTP for SHI in Ethiopia might be because of the negative attitude of the population in the formal sector toward the scheme. On the other hand, it could be because the premium is beyond the ability of the majority to pay (45). For instance, in Nigeria, of the 82% who agreed to pay, only 65% of the households had the ability to pay the average premium (40). In order to design the scheme in a way that will be practical, socially acceptable, and economically viable while also meeting the demands of the population in the formal sector both in the present and in the future, it may be a good idea to consider the sociocultural, economic, and political environments (46).

In research carried out in various countries, socioeconomic characteristics were reported to affect people's WTP for health insurance programs. Accordingly, the WTP for SHI was found to be influenced by age (41, 44, 47), gender (40, 42, 44, 48), level of education (40, 42, 44, 47, 49–52), residence (40, 41), household size (40, 44, 47–49, 52), occupational status (40, 49), household income (40, 47, 49, 51), and marital status (50). Thus, it might be important to consider the socio-demographic diversity of the population in the formal sector while implementing SHI.

The health and illness statuses of the households, including self-rated healthiness and the presence of acute and chronic illnesses, were the other factors influencing the WTP for SHI. The presence of illness was also reported to be a major factor affecting the WTP for the scheme in Nepal (44), Vietnam (53), and Saudi Arabia (42).

Health service related factors such as previous medical bills, referral systems, regular medical checkups, and health service quality were also reported to be important determinants of the WTP for SHI. Similarly, in Bangladesh, the number of visits to the doctor was found to play a key role in determining the WTP (41). In Nepal, quality services were an important determinant for the decision to pay for the scheme (44). In Mongolia, past or current medical expenditures were significantly associated with WTP (51). In Nigeria, the mode of payment for healthcare was reported to be an important predictor for the WTP for the scheme (50).

Furthermore, awareness, knowledge, perception, attitude, and factors related to the scheme, such as trust, premium amount, and benefit packages, were reported to be influential factors in the WTP for SHI. Likewise, in Nepal (44) and Vietnam (53), awareness level and knowledge of the scheme were significantly associated with the WTP for SHI program (44). In fact, the more people who know about SHI, the higher their WTP (53).

Since the premium amount may need to be subsidized by beneficiaries, it is important to consider differences between the WTP and the cost of the benefits package to be offered (40). It is also good not to rely on households' premiums as a major financing source and to increase the government's fiscal capacity for an equitable health care system using other sources (47). This is because relying solely on SHI schemes to achieve UHC may not be plausible (54). Integrating the poor into SHI will require the strengthening of institutions and an increase in political will to effectively implement exemption policies across all sectors of the economy (55).

Because determining the health insurance premium is the most important aspect of providing SHI (41), a shared strategic vision for a single mandatory health insurance, collaboration with diverse stakeholders in the implementation of the scheme, enhanced monitoring of transparency, unlimited involvement of the private sector in service delivery, and strong accountability of the government or insurer are also equally important (56).

Ceteris paribus, it was found that the health professionals were less likely to pay for the scheme. Hence, since they are the central players on the supply side of the scheme, the issue seems to be worrying, dictating that more effort or a different strategy is needed in this regard.

### Policy and practical implications

Because they must pay for health bills out of their own pockets, about 100 million individuals are forced into extreme poverty every year (57, 58), which mandates governments to design and implement effective strategies to secure their citizens from such catastrophes. The scenario is not different for Ethiopia. However, catastrophic health spending represents a sufficient but not a necessary condition for financial hardship to occur. This is because financial hardship monitoring also relies on household budget surveys, household income and expenditure surveys, household living standard surveys, or socioeconomic surveys (59). Moreover, because the Bismarck model associates the right to healthcare with employment through mandatory payroll deductions, committed compulsory implementation might be equally important (4).

### Limitations

The direction of association between the dependent variable (WTP for SHI) and the independent variables was not estimated due to the variability of the reports of the included studies. Thus, the variables or factors were reported using qualitative synthesis. Studies other than English were not included. The data was pooled despite high heterogeneity.

## Conclusion

The WTP for SHI in Ethiopia was found to be < 50% and was found to be influenced by sociodemographic factors, health and illness status, health service related factors, awareness or knowledge, perception, attitude, and factors related to the scheme. Keeping all other variables constant, the health professionals were less interested in paying for the scheme than teachers. Thus, a further nation-wide study, based on profession or occupation, that will investigate the WTP of the workers in the formal sector for SHI and their concerns seems essential through qualitative and quantitative approaches.

## Author contributions

EMB and HNT conceived and designed the review, supervised and performed the review, extracted, analyzed and interpreted the data, wrote the paper, and contributed to the writing and reviewing of the manuscript. BBC conceived and designed the review, extracted and performed the review, analyzed and interpreted the data, and wrote and reviewed the manuscript. MHK and BDW conceived and designed the review, supervised the review, analyzed and interpreted the data, and reviewed the manuscript. All authors reviewed and approved the final version of the manuscript.

## Supplementary material

Supplementary material 1 PRISMA 2020 Checklist.

Supplementary material 2 Database search strategy.
