# Corrigendum: Willingness to pay for social health insurance in Ethiopia: a systematic review and meta-analysis

**DOI:** 10.3389/fpubh.2023.1252289

**Published:** 2023-09-27

**Authors:** Ewunetie Mekashaw Bayked, Husien Nurahmed Toleha, Beletu Berihun Chekole, Birhanu Demeke Workneh, Mesfin Haile Kahissay

**Affiliations:** ^1^Department of Pharmacy, College of Medicine and Health Sciences (CMHS), Wollo University, Dessie, Ethiopia; ^2^Department of Pharmacy, Woldia Comprehensive Specialized Hospital, Woldia, Ethiopia; ^3^Department of Pharmaceutics and Social Pharmacy, School of Pharmacy, College of Health Sciences, Addis Ababa University, Addis Ababa, Ethiopia

**Keywords:** willingness to pay, social health insurance, associated factors, systematic review, meta-analysis, Ethiopia

In the published article, there was an error in the *Results* section of the **Abstract**. This error occurred when we compared the willingness of teachers and health professionals to pay for social health insurance, without considering the proportions (3.22:0.42) of the odds ratios of the two groups' willingness to pay.

A correction has been made to the **Abstract**, *Results*. This sentence previously stated:

“Professionally, teachers were 3.22 times more likely to pay for the scheme (OR = 3.22; 95% CI: 1.80–5.76) than health professionals.”

The corrected sentence appears below:

“Professionally, teachers were 7.67 times more likely to pay for the scheme (OR = 3.22; 95% CI: 1.80–5.76) than health professionals (OR = 0.42; 95% CI: 0.19–0.93).”

In the published article, there was an error in the **Results** section, “*Results of Quantitative Synthesis”*, paragraph 3. The error occurred for the same reason stated above in the previous correction.

A correction has been made to **Results**, “*Results of Quantitative Synthesis”*, paragraph 3. This sentence previously stated:

“Thus, as shown in Figure 5, regarding the sub-group analysis by profession, teachers were 3.22 times more likely to pay for SHI (OR = 3.22; 95% CI: 1.80–5.76) than health professionals.”

The corrected sentence appears below:

“Thus, as shown in Figure 5, regarding the sub-group analysis by profession, teachers were 7.67 times more likely to pay for SHI (OR = 3.22; 95% CI: 1.80–5.76) than health professionals (OR = 0.42; 95% CI: 0.19–0.93).”

For clarity, the sentence: “However, the WTP of health professionals to the scheme was 58% less likely (OR = 0.42; 95% CI: 0.19–0.93) than that of teachers”, which is the last sentence in paragraph 3 of the **Results** section, subsection “*Results of Quantitative Synthesis”*, has been removed.

In the published article, there was an error in the **Discussion** section, subsection “*Limitations”*. This was a typographical error.

A correction has been made to **Discussion**, “*Limitations”*. The sentence previously stated:

“The direction of association between the dependent variable (WTP for SHI) and the dependent variables was not estimated due to the variability of the reports of the included studies.”

The corrected sentence appears below:

“The direction of association between the dependent variable (WTP for SHI) and the independent variables was not estimated due to the variability of the reports of the included studies.”

In the published article, there was an error in Table 2 as published. Under the column “*Statistical Method”*, the abbreviations “M-H” in each row should be corrected as “IV” as they appear in the pooled result.

The corrected Table 2 and its caption appear below:

**Table 2:** Pooled result of the meta-analysis by region.

**Subgroup**	**Studies**	**Participants**	**Events**	**Percent**	**Statistical method**	**Effect estimate**
Amhara	5	2,796	1,149	41.10	Odds ratio (IV, Random, 95% CI)	0.72 [0.33, 1.58]
Tigray	3	1,620	809	49.94	Odds ratio (IV, Random, 95% CI)	1.40 [0.43, 4.58]
Addis Ababa	6	2,403	980	40.78	Odds ratio (IV, Random, 95% CI)	0.72 [0.30, 1.73]
SNNPR	2	1,020	286	28.04	Odds ratio (IV, Random, 95% CI)	0.43 [0.01, 18.06]
Oromia	1	275	76	27.64	Odds ratio (IV, Random, 95% CI)	0.38 [0.29, 0.50]
Harar	1	272	243	89.34	Odds ratio (IV, Random, 95% CI)	8.38 [5.71, 12.31]
**Pooled result**	**18**	**8,386**	**3,543**	**42.25**	**Odds ratio (IV, Random, 95% CI)**	**0.84 [0.52, 1.36]**

The authors apologize for these errors and state that this does not change the scientific conclusions of the article in any way. The original article has been updated.

