# Supplementary material for: Willingness to pay for social health insurance in Ethiopia: A systematic review and meta-analysis
Source: Front Public Health. 2023 Mar 22;11:1089019. doi: 10.3389/fpubh.2023.1089019 (PMC10073487; doi:10.3389/fpubh.2023.1089019)
Supplement: Supplementary material 2 — Database search strategy. [file Table_2.DOCX]

**Supplementary file 2. Database search strategy**

1. **PubMed (n = 11):**

Search: **(((Willingness to pay) AND (social health insurance)) AND (Ethiopia)**

"Willingness"[All Fields] AND "pay"[All Fields] AND (("social behavior"[MeSH Terms] OR ("social"[All Fields] AND "behavior"[All Fields]) OR "social behavior"[All Fields] OR "sociality"[All Fields] OR "social"[All Fields] OR "socialisation"[All Fields] OR "socialization"[MeSH Terms] OR "socialization"[All Fields] OR "socialise"[All Fields] OR "socialised"[All Fields] OR "socialising"[All Fields] OR "socialities"[All Fields] OR "socializations"[All Fields] OR "socialize"[All Fields] OR "socialized"[All Fields] OR "socializers"[All Fields] OR "socializes"[All Fields] OR "socializing"[All Fields] OR "socially"[All Fields] OR "socials"[All Fields]) AND ("insurance, health"[MeSH Terms] OR ("insurance"[All Fields] AND "health"[All Fields]) OR "health insurance"[All Fields] OR ("health"[All Fields] AND "insurance"[All Fields]))) AND ("ethiopia"[MeSH Terms] OR "ethiopia"[All Fields] OR "ethiopia s"[All Fields])

**Translations**

**social:** "social behavior"[MeSH Terms] OR ("social"[All Fields] AND "behavior"[All Fields]) OR "social behavior"[All Fields] OR "sociality"[All Fields] OR "social"[All Fields] OR "socialisation"[All Fields] OR "socialization"[MeSH Terms] OR "socialization"[All Fields] OR "socialise"[All Fields] OR "socialised"[All Fields] OR "socialising"[All Fields] OR "socialities"[All Fields] OR "socializations"[All Fields] OR "socialize"[All Fields] OR "socialized"[All Fields] OR "socializers"[All Fields] OR "socializes"[All Fields] OR "socializing"[All Fields] OR "socially"[All Fields] OR "socials"[All Fields]

**health insurance:** "insurance, health"[MeSH Terms] OR ("insurance"[All Fields] AND "health"[All Fields]) OR "health insurance"[All Fields] OR ("health"[All Fields] AND "insurance"[All Fields])

**Ethiopia:** "ethiopia"[MeSH Terms] OR "ethiopia"[All Fields] OR "ethiopia's"[All Fields]

1. **Research4Life (R4L) - Hinari (N= 21):**

**Search:** (Willingness to pay) AND (Social health insurance) AND (Ethiopia)

Selected by:

**Content Type:** Any type

**Discipline:** Any type

**Language:** English

**Limit to:** Items with full text online

1. **Google scholar (n = 17)** **– using “Perish or Publish” software:**

**Willingness to pay social health insurance Ethiopia [title]**

*Publish or Perish 8.4.4041.8250 (basic report)
WinPosix (x64) edition, running on WinPosix 10.0.19042 (x64)*

**Search terms**

**Title words:** Willingness to pay social health insurance Ethiopia
**Years:** all
**Other options:** include citations; include patents

**Data retrieval**

**Data source:** Google Scholar
**Search date:** 2022-09-02 00:56:37 +0300
**Cache date:** 2022-09-02 00:56:41 +0300
**Search result:** [0] No error

***Important:*** *This data source provides only abbreviated data. Any ellipses (... marks) shown in this report originate with the data source; they are NOT caused by subsequent processing in Publish or Perish.*

**Metrics**

**Reference date:** 2022-09-02 00:56:41 +0300
**Publication years:** 2014-2022
**Citation years:** 8 (2014-2022)
**Papers:** 17
**Citations:** 120
**Citations/year:** 15.00 (acc1=6, acc2=5, acc5=3, acc10=1, acc20=0)
**Citations/paper:** 7.06
**Authors/paper:** 3.35/3.0/4 (mean/median/mode)
**Age-weighted citation rate:** 31.53 (sqrt=5.62), 8.73/author
**Hirsch h-index:** 4 (a=7.50, m=0.50, 106 cites=88.3% coverage)
**Egghe g-index:** 10 (g/h=2.50, 119 cites=99.2% coverage)
**PoP hI,norm:** 3
**PoP hI,annual:** 0.38
**Fassin hA-index:** 4

**Results**

MT Gidey, GB Gebretekle, ME Hogan, ... (2019) **Willingness to pay for social health insurance and its determinants among public servants in Mekelle City, Northern Ethiopia: a mixed methods study**. *Cost Effectiveness and …*, Springer, doi:10.1186/s12962-019-0171-x, cited by 35 (11.67 per year)

TA Agago, M Woldie, S Ololo (2014) **Willingness to join and pay for the newly proposed social health insurance among teachers in Wolaita Sodo town, South Ethiopia**. *Ethiopian journal of health sciences*, ajol.info, cited by 50 (6.25 per year)

A Mekonne, B Seifu, C Hailu, A Atomsa (2020) **Willingness to pay for social health insurance and associated factors among health care providers in Addis Ababa, Ethiopia**. *BioMed Research …*, hindawi.com, cited by 10 (5.00 per year)

A Setegn, G Andargie, G Amare, ... (2021) **Willingness to pay for social health insurance among teachers at governmental schools in Gondar town, Northwest Ethiopia**. *Risk Management and …*, ncbi.nlm.nih.gov, cited by 4 (4.00 per year)

MA Kokebie, ZA Abdo, S Mohamed, ... (2022) **Willingness to pay for social health insurance and its associated factors among public servants in Addis Ababa, Ethiopia: a cross-sectional study**. *BMC Health Services …*, Springer, doi:10.1186/s12913-022-08304-8

F Mekonnen Degie, Y Agumas Ambelie, ... (2021) **Willingness to Pay for Social Health Insurance and Its Predictors among Government Employees in Mujja Town, Ethiopia**. *The Scientific World …*, hindawi.com, cited by 1 (1.00 per year)

AT Gessesse, AA Berhe, MG Tilahun, TW Teklemariam (2016) **Factors Associated with Willingness to Pay for Social Health Insurance Among Government Employees in Tigrai Region, Northern Ethiopia**., eajahme.com, cited by 2 (0.33 per year)

SG Hailu, GA Firde, AT Debele, HF Zakaria, BT Merga (2022) **Teachers' willingness to pay for social health insurance and its determinant factors at Harar region, Ethiopia, 2021**., researchsquare.com

Z Regassa, E Negera, T Silashi, Z Kaba (2022) **Willingness to join and pay social health insurance and associated factors among public sectors workers in Didu Woreda, South West Ethiopia, 2018**., researchsquare.com

WN Mekonnen, M Wondaferew, AB Mekonen (2019) **Willingness to Join and Pay for Social Health Insurance Scheme Among employees in Debere Berhan Town, Ethiopia**., researchsquare.com

B Mulatu, A Mekuria, B Tassew (2020) **Willingness to join and pay for Social Health Insurance among Public Servants in Arba Minch town, Gammo Zone, Southern Ethiopia**., researchsquare.com, cited by 1 (0.50 per year)

D DEMELASH (2022) **WILLINGNESS TO JOIN AND PAY FOR SOCIAL HEALTH INSURANCE AND ASSOCIATED FACTORS AMONG CIVIL SERVANTS IN MERAWI TOWN …**.

EM Amilaku, FW Fentaye, AM Mekonen, ... (2022) **Willingness to pay for social health insurance among public civil servants: A cross-sectional study in Dessie City Administration, North-East Ethiopia**. *… in Public Health*, ncbi.nlm.nih.gov

Y Lasebew, Y Mamuye, S Abdelmenan (2017) **Willingness to pay for the newly proposed social health insurance among health workers at St. Paul's Hospital Millennium Medical College, Addis Ababa …**. *Int J Health Econ Policy*, cited by 11 (2.20 per year)

M LasebewY **AbdelmenanS.(2017). Willingness to Pay for the Newly Proposed Social Health Insurance among Health Workers at St. Paul's Hospital Millennium …**. *International Journal of Health Economics and Policy*, cited by 2 (0.00 per year)

A Defar, A Seyum, T Gelibo, T Getachew (2016) **Willingness to pay for social health insurance and associated factors among health sector employees in Addis Ababa, Ethiopia**. *MOH 18th Annual Review Meeting. MOH*, cited by 2 (0.33 per year)

AA Tesfamichael, W Mirkuzie, O Shimeles (2014) **Willingness to join and pay for the newly proposed social health insurance among teachers in Wolaita Sodo town, south Ethiopia Ethiop J Health Sci**., cited by 2 (0.25 per year)

1. **Scopus (n =** **7) – using “perish or publish” software:**

**Willingness to pay social health insurance Ethiopia [title]**

*Publish or Perish 8.4.4041.8250 (basic report)
WinPosix (x64) edition, running on WinPosix 10.0.19042 (x64)*

**Search terms**

**Title words:** Willingness to pay social health insurance Ethiopia
**Years:** all

**Data retrieval**

**Data source:** Scopus
**Search date:** 2022-09-02 00:56:15 +0300
**Cache date:** 2022-09-02 00:56:17 +0300
**Search result:** [0] No error

***Important:*** *This data source returns only one author per article; this affects the calculation of per-author metrics.*

**Metrics**

**Reference date:** 2022-09-02 00:56:17 +0300
**Publication years:** 2014-2022
**Citation years:** 8 (2014-2022)
**Papers:** 7
**Citations:** 51
**Citations/year:** 6.38 (acc1=5, acc2=4, acc5=0, acc10=0, acc20=0)
**Citations/paper:** 7.29
**Authors/paper:** 1.00/1.0/1 (mean/median/mode)
**Age-weighted citation rate:** 15.71 (sqrt=3.96), 15.71/author
**Hirsch h-index:** 4 (a=3.19, m=0.50, 50 cites=98.0% coverage)
**Egghe g-index:** 7 (g/h=1.75, 51 cites=100.0% coverage)
**PoP hI,norm:** 4
**PoP hI,annual:** 0.50
**Fassin hA-index:** 3

**Results**

T.A.l. Agago (2014) **Willingness to join and pay for the newly proposed social health insurance among teachers in Wolaita Sodo Town, South Ethiopia**. *Ethiopian journal of health sciences* 24(3), pp. 195-202, ISSN 1029-1857, doi:10.4314/ejhs.v24i3.2, cited by 27 (3.38 per year)

M.T. Gidey (2019) **Willingness to pay for social health insurance and its determinants among public servants in Mekelle City, Northern Ethiopia: A mixed methods study**. *Cost Effectiveness and Resource Allocation* 17(1), ISSN 1478-7547, doi:10.1186/s12962-019-0171-x, cited by 13 (4.33 per year)

A. Mekonne (2020) **Willingness to Pay for Social Health Insurance and Associated Factors among Health Care Providers in Addis Ababa, Ethiopia**. *BioMed Research International* 2020, ISSN 2314-6133, doi:10.1155/2020/8412957, cited by 6 (3.00 per year)

A. Setegn (2021) **Willingness to pay for social health insurance among teachers at governmental schools in Gondar Town, Northwest Ethiopia**. *Risk Management and Healthcare Policy* 14, pp. 861-868, ISSN 1179-1594, doi:10.2147/RMHP.S298256, cited by 4 (4.00 per year)

F. Mekonnen Degie (2021) **Willingness to Pay for Social Health Insurance and Its Predictors among Government Employees in Mujja Town, Ethiopia**. *Scientific World Journal* 2021, ISSN 2356-6140, doi:10.1155/2021/3149289, cited by 1 (1.00 per year)

E.M. Amilaku (2022) **Willingness to pay for social health insurance among public civil servants: A cross-sectional study in Dessie City Administration, North-East Ethiopia**. *Frontiers in Public Health* 10, ISSN 2296-2565, doi:10.3389/fpubh.2022.920502

M.A. Kokebie (2022) **Willingness to pay for social health insurance and its associated factors among public servants in Addis Ababa, Ethiopia: a cross-sectional study**. *BMC Health Services Research* 22(1), ISSN 1472-6963, doi:10.1186/s12913-022-08304-8

1. **Semantic Scholar (n = 10) – using “perish or publish” software:**

**Willingness to pay for social health insurance in Ethiopia**

*Publish or Perish 8.4.4041.8250 (basic report)
WinPosix (x64) edition, running on WinPosix 10.0.19042 (x64)*

**Search terms**

**Keywords:** Willingness to pay for social health insurance in Ethiopia

**Data retrieval**

**Data source:** Semantic Scholar
**Search date:** 2022-09-02 00:57:27 +0300
**Cache date:** 2022-09-02 00:57:29 +0300
**Search result:** [0] No error

***Important:*** *The Semantic Scholar API is still under development and only provides limited paper search options and limited result data.*

**Metrics**

**Reference date:** 2022-09-02 00:57:29 +0300
**Publication years:** 2014-2022
**Citation years:** 8 (2014-2022)
**Papers:** 10
**Citations:** 84
**Citations/year:** 10.50 (acc1=5, acc2=4, acc5=2, acc10=0, acc20=0)
**Citations/paper:** 8.40
**Authors/paper:** 3.80/4.0/4 (mean/median/mode)
**Age-weighted citation rate:** 24.04 (sqrt=4.90), 6.48/author
**Hirsch h-index:** 4 (a=5.25, m=0.50, 80 cites=95.2% coverage)
**Egghe g-index:** 9 (g/h=2.25, 84 cites=100.0% coverage)
**PoP hI,norm:** 2
**PoP hI,annual:** 0.25
**Fassin hA-index:** 4

**Results**

Atalele Setegn, G. Andargie, Getasew Amare, Ayal Debie (2021) **Willingness to Pay for Social Health Insurance Among Teachers at Governmental Schools in Gondar Town, Northwest Ethiopia**., doi:10.2147/RMHP.S298256, cited by 4 (4.00 per year)

Feleku Mekonnen Degie, Yeshambel Agumas Ambelie, Yared Mulu Gelaw, Getahun Fentaw Mulaw, Fentaw Wassie Feleke (2021) **Willingness to Pay for Social Health Insurance and Its Predictors among Government Employees in Mujja Town, Ethiopia**., doi:10.1155/2021/3149289, cited by 1 (1.00 per year)

Meles Tekie Gidey, G. B. Gebretekle, M. Hogan, T. G. Fenta (2019) **Willingness to pay for social health insurance and its determinants among public servants in Mekelle City, Northern Ethiopia: a mixed methods study**., doi:10.1186/s12962-019-0171-x, cited by 25 (8.33 per year)

Abel Mekonne, Benyam Seifu, Chernet Hailu, A. Atomsa (2020) **Willingness to Pay for Social Health Insurance and Associated Factors among Health Care Providers in Addis Ababa, Ethiopia**., doi:10.1155/2020/8412957, cited by 8 (4.00 per year)

Eshetie Meseret Amilaku, Fasil Walelign Fentaye, Asnakew Molla Mekonen, E. M. Bayked (2022) **Willingness to pay for social health insurance among public civil servants: A cross-sectional study in Dessie City Administration, North-East Ethiopia**., doi:10.3389/fpubh.2022.920502

Ataklti Gessesse, A. Berhe, Mulugeta Tilahun, Tesfay Teklemariam (2020) **Factors Associated with Willingness to Pay for Social Health Insurance among Government Employees in Tigrai Region, Northern Ethiopia**., cited by 1 (0.50 per year)

Melkamu Ayalew Kokebie, Ziyad Ahmed Abdo, Shikur Mohamed, Belayneh Leulseged (2022) **Willingness to pay for social health insurance and its associated factors among public servants in Addis Ababa, Ethiopia: a cross-sectional study**., doi:10.1186/s12913-022-08304-8

Bahiru Mulatu, Aleme Mekuria, Berhan Tassew (2020) **Willingness to join and pay for Social Health Insurance  among Public Servants in Arba Minch town, Gammo Zone, Southern Ethiopia**., doi:10.21203/rs.3.rs-22331/v1, cited by 1 (0.50 per year)

W. Mekonnen, Mesfin Wondaferew, Adugnaw Birhane Mekonen (2019) **Willingness to Join and Pay for Social Health Insurance Scheme Among employees in Debere Berhan Town, Ethiopia**., doi:10.21203/rs.2.15992/v1, cited by 1 (0.33 per year)

T. A. Agago, M. Woldie, S. Ololo (2014) **Willingness to Join and Pay for the Newly Proposed Social Health Insurance among Teachers in Wolaita Sodo Town, South Ethiopia**., doi:10.4314/EJHS.V24I3.2, cited by 43 (5.38 per year)
